# Risk factors for post-hemorrhagic hydrocephalus among infants with intraventricular hemorrhage

**DOI:** 10.1186/2045-8118-12-S1-O18

**Published:** 2015-09-18

**Authors:** Hannah M Tully, Christopher M Traudt, Tessa Rue, Assaf P Oron, Tamara D Simon, Walter A Kukull, Dan Doherty

**Affiliations:** 1 Seattle Children's Research Institute, USA; 2 Seattle Children's Hospital, USA; 3 Univeristy of Washington, USA

## Introduction

Post-hemorrhagic hydrocephalus (PHH) is a common complication of intraventricular hemorrhage (IVH) of prematurity, but the risk factors that predispose infants with IVH to PHH have not been fully elucidated.

## Methods

Retrospective cohort study of infants diagnosed with IVH between 2004 and 2014, with and without PHH, from the Pediatric Hospital Information System, which contains data from 44 American children's hospitals. Poisson regression was used to calculate adjusted risk ratios (RRs) and 95% confidence intervals (CIs) for multiple potential risk factors, stratified by IVH grade (I/II, III/IV).

## Results

Among 19,077 infants with IVH who lived at least 60 days, 2,422 (12.7%) developed PHH, including 233 (1.9%) of 12,078 infants with Grade I/II IVH and 1,917 (37.1%) of 5,165 infants with Grade III/IV IVH. Among the infants with Grade III/IV IVH, Hispanic and Asian ethnicity were associated with a reduced risk of PHH compared to whites (RR: 0.84, 95%CI: 0.75, 0.93; RR: 0.63, 95%CI: 0.43, 0.92, respectively). Meningitis was associated with an increased risk of PHH (RR: 2.42, 95%CI: 2.25, 2.61). Patent ductus arteriosus was associated with a reduced risk, (RR: 0.80, 95%CI: 0.75, 0.86), as was use of the NSAID indomethacin, used to treat PDA (RR: 0.67, 95%CI: 0.60, 0.76). Atropine and dexamethasone were associated with increased risk (RR: 1.48, 95%CI: 1.38, 1.59; RR: 1.14, 95%CI: 1.06, 1.23). Pressors, nitric oxide and steroids (other than dexamethasone) were all associated with reduced risk (RR: 0.64, 95%CI: 0.60, 0.69; RR: 0.69, 95%CI: 0.55, 0.86; RR: 0.71, 95%CI: 0.64, 0.79).

## Conclusions

Several innate, acquired and potentially modifiable exposures appear to influence the development of PHH among infants with IVH. The reduced risk associated with certain classes of medication such as pressors, NSAIDs and steroids warrants further investigation.

